# Distinct protein and metabolic profiles in patients with advanced clear-cell renal cell carcinoma treated with sunitinib: a study of the Spanish oncology genitourinary group

**DOI:** 10.3389/fonc.2026.1639330

**Published:** 2026-04-29

**Authors:** Guillermo Quintás, Elena Sanmartín, Alicia Garcia-Gimenez, José Muñoz-Langa, Aida Collado, Cristina Suárez, Xavier García del Muro, María José Méndez-Vidal, José García-Sánchez, Carmen Salvador-Coloma, Nuria Laínez, Enrique Gallardo, Javier Munárriz, Sergio Vázquez, Carmen Molins, Jaime Font de Mora, Gaspar Reynés

**Affiliations:** 1 Health and Biomedicine, Leitat Technological Center, Terrassa, Spain; 2 Centro de Investigación Biomédica en Red de Enfermedades Hepáticas y Digestivas (CIBERehd), Instituto de Salud Carlos III, Madrid, Spain; 3 Direcció General de Salut Pública de València, Laboratori de Microbiologia, València, Spain; 4 Department of Medical Oncology, Hospital Universitari i Politècnic La Fe, Valencia, Spain; 5 Medical Oncology, Vall d´Hebron Institute of Oncology (VHIO), Hospital Universitari Vall d´Hebron, Vall d´Hebron Barcelona Hospital Campus, L'Hospitalet de Llobregat, Spain; 6 Catalan Institute of Oncology, IDIBELL, University of Barcelona, L’Hospitalet de Llobregat, Barcelona, Spain; 7 Department of Medical Oncology, Maimonides Institute for Biomedical Research of Córdoba, Hospital Reina Sofía, Córdoba, Spain; 8 Department of Medical Oncology, Hospital Arnau de Vilanova, Valencia, Spain; 9 Department of Medical Oncology, Complejo Hospitalario de Navarra, Pamplona, Navarra, Spain; 10 Department of Medical Oncology, Parc Taulí Hospital Universitari, Institut d’Investigació i Innovació, Parc Taulí I3PT, Universitat Autònoma de Barcelona, Sabadell, Spain; 11 Department of Medical Oncology, Hospital Provincial de Castelló, Castelló de la Plana, Castellón, Spain; 12 Department of Medical Oncology, Hospital Universitario Lucus Augusti, Lugo, Spain; 13 Department of Medical Oncology, Hospital Universitario Doctor Peset, Valencia, Spain; 14 Group of Clinical and Translational Research in Cancer, Health Research Institute Hospital La Fe, Valencia, Spain

**Keywords:** ARG-1, clear cell renal cell carcinoma, extreme discordant phenotypes, IL-6, S100A9, sunitinib, tryptophan metabolism

## Abstract

**Background:**

New biomarkers are needed to improve treatment selection in patients with metastatic clear cell renal cell carcinoma (CCRCC). This study aims to identify predictive biomarkers through the investigation of serum proteins related to angiogenesis and tumor immune escape, alongside metabolic patterns associated with clinical outcomes.

**Methods:**

We conducted a prospective, multicenter study in patients with locally advanced or metastatic CCRCC receiving first-line sunitinib. Serum protein levels, including those related to angiogenesis and immune escape, were measured. Additionally, untargeted lipidomic and targeted metabolic analyses focused on tryptophan metabolism and amino acid profiles were performed. Routine blood test results were recorded, and correlations among all parameters were analyzed.

**Results:**

Thirty-eight patients from ten Spanish hospitals were included. Median progression-free survival (PFS) was 9.87 months, and median overall survival (OS) was 21.10 months. Multivariate analysis identified higher interleukin-6 (IL-6), arginase-1, and S100 calcium binding protein A9 (S100A9) concentrations as significant predictors of shorter OS. We defined three distinct risk groups based on the combined levels of S100A9 and IL-6: high levels of both proteins, high levels of either one protein, or low levels of both proteins confer a poor, intermediate and favorable prognosis, respectively. Metabolomic analysis revealed significant differences in the tryptophan-kynurenine pathway between patients with extreme PFS and OS phenotypes. Elevated levels of tryptophan metabolites were associated with poorer PFS, while alterations in amino acids and tryptophan metabolites correlated with OS extremes. Notably, significant correlations were observed between IL-6 levels and increased tryptophan metabolism.

**Conclusions:**

This study underscores the prognostic value of specific proteins and metabolites in metastatic CCRCC, proposing potential biomarkers for patient stratification and treatment response prediction.

## 1 Introduction

Kidney cancer is the ninth most common tumor in men and the fourteenth most common tumor in women worldwide, accounting for 214,000 and 124,000 cases per year, respectively. A 15-fold variation in incidence rates is observed by geographic area, with higher rates in developed countries (1). This significant variation suggests a link between lifestyle factors and kidney cancer incidence.

Kidney cancer is a family of metabolic diseases. Each type of renal cell carcinoma (RCC) is associated with specific genetic abnormalities that interact with specific cell metabolic pathways (2). Hypoxia-inducible factor (HIF)-1α and HIF-2α are transcription factors that regulate genes that lead to cell adaptation to hypoxia. Clear cell renal cell carcinoma (CCRCC) accounts for 75% of kidney cancers (3). CCRCC is associated with von Hippel-Lindau (VHL) gene mutations, which prevent the ubiquitination of HIF-1α and HIF-2α. Stabilized HIF1α and HIF2α increase red blood cell production by upregulating erythropoietin, angiogenesis via vascular endothelial growth factors (VEGFs), and cellular growth by upregulating platelet-derived growth factor (PDGF) and transforming growth factor α (TGFA α) (4).

Metabolic alterations lead cells to use aerobic glycolysis instead of mitochondrial oxidative phosphorylation to generate energy. This mechanism, known as the Warburg effect (5), although less efficient for energy production, provides cells with elements needed for proliferation, such as nucleotides, lipids, and amino acids. Tryptophan is an essential amino acid that, in addition to its role in protein synthesis, is metabolized through three main pathways: decarboxylation to produce tryptamine, hydroxylation to produce serotonin, and the kynurenine pathway mediated by the enzymes indoleamine 2,3-dioxygenase-1 (IDO-1), IDO-2, and tryptophan 2,3-dioxygenase (TDO), which are transcribed only in the liver. During the first phase of cancer immunoediting, IDO-1, produced at low levels by antigen-presenting cells, depletes tryptophan from tumor cells and inhibits their proliferation. When the equilibrium phase progresses to the escape phase, large amounts of IDO-1 are produced by tumor cells and immune cells recruited to the tumor microenvironment. The deprivation of tryptophan, as well as elevated concentrations of tryptophan metabolites such as kynurenine, 3-hydroxykynurenine, and 3-hydroxyanthranilic acid, hinders effector T cell and natural killer cell functions (6–8). IDO is also involved in recruiting T regulatory cells (Tregs) that suppress the immune response (9–11). A higher kynurenine/tryptophan ratio has been associated with poor prognosis in CCRCC patients (12). In patients with RCC treated with nivolumab, an anti-Programmed cell death protein-1 (PD-1) treatment, kynurenine concentrations and the kynurenine/tryptophan ratio increase significantly in a subset of patients and correlate with worse survival (13). Lipid metabolism is also crucial in RCC. Obesity is one of the leading risk factors for developing kidney cancer. However, overweight and obese patients are more likely to present with less aggressive tumors than normal-weight patients are (14). Polyunsaturated fatty acids play a crucial role in tumor-related immunosuppression and promote the expansion of myeloid-derived suppressor cells (MDSCs) (15).

Overall, these results highlight the fundamental role of studying proteins and metabolomics in shaping therapeutic intervention strategies for RCC in real-world medical settings (16). This is an active field of research (17–21).

In this prospective study, we explored the impact of serum levels of proteins related to angiogenesis and immunosuppression on the prognosis of patients with metastatic CCRCC who received the TKI sunitinib as first-line treatment. VEGF-A, tissue factor (TF), interleukin-6 (IL-6), interleukin-8 (IL-8), angiopoietin-2 (Ang-2), S100 bindingA9 (S100A9), tumor necrosis factor α (TNF-α), and arginase-1 were included in the study. VEGF-A, the most relevant VEGF involved in tumor-induced angiogenesis, also leads to the overexpression of Programmed cell death protein 1 (PD-1) and other inhibitory checkpoints involved in CD8+ T-cell inactivation. Interestingly, this effect is reversed by sunitinib, a tyrosine kinase inhibitor (TKI) targeting the VEGF-A–VEGFR axis, which is widely used for CCRCC treatment (22). Tissue factor increases angiogenesis via expression of VEGF and IL-8. Moreover, TF protects tumor cells from natural killer cells (23). IL-6 promotes the amplification and immunosuppressive function of MDSCs through the induction of suppressor of cytokine signaling 3 (SOCS3) dysfunction and activation of the Janus kinase/signal transducer and activator of transcription (JAK/STAT) signaling pathway (24). IL-8, also known as CXCL8, is a chemokine with pro-inflammatory and proangiogenic activity. Cancer-derived IL-8 is a chemoattractant for MDSCs (25). Angiopoietin ligands stimulate angiogenesis and control vascular remodeling and maturation through endothelial TIE receptors, TIE1 and TIE2. Ang2 might facilitate the metastatic process by disrupting pericyte attachement to the endotelial wall (26). S100A9 is involved in tumor proliferation, invasion, and metastasis, and enhances premetastatic niche formation (27). Tumor-induced S100A9 elicits the accumulation and activation of MDSCs (28–30), which impairs the ability of the immune system to eliminate tumor cells. MDSCs secrete Arg-1, which hinders T cell proliferation and function through depletion of L-arginine (31). Increased levels of Arg-1 and low levels of L-arginine were found in patients with RCC compared with healthy controls (32). Activation of HIF-1α and other transcription factors stimulates the production of TNF-α, which recruits and activates inflammatory cells (33). Transmembrane TNFα has been shown to increase the immunosuppressive activity of MDSCs by increasing the transcription of ARG-1 and iNOS (34).

We also established correlations among the proteins studied and between these proteins and various routine blood test parameters. Additionally, we evaluated the serum metabolic profiles associated with extreme discordant prognostic phenotypes (35) using complementary targeted and untargeted liquid chromatography-mass spectrometry (LC-MS) methods.

## 2 Methods

### 2.1 Study design

We conducted a prospective, multicenter study in patients with locally advanced or metastatic CCRCC receiving first-line sunitinib, a tyrosine kinase inhibitor targeting the VEGF-A/VEGFR axis. The aim of this study was to explore the prognostic value of circulating proteins related to angiogenesis and immune escape and to identify metabolic patterns associated with extreme discordant RCC phenotypes. The study was conducted according to the guidelines of the Declaration of Helsinki, and approved by the Clinical Research Ethical Committees of all participating centers (Protocol SOG-ITQ-2013-01). All patients signed a written informed consent.

### 2.2 Patient selection

Patients were prospectively recruited from ten Spanish hospitals from February 2014 to April 24, 2015. The study was closed on May 21, 2015. All patients signed a written informed consent. The study was approved by the Institutional Ethics Committees of all participating centers (Protocol SOG-ITQ-2013-01). The study was carried out in accordance with the Declaration of Helsinki.

Additional inclusion criteria were: the absence of other malignancies; Eastern Cooperative Oncology Group (ECOG) performance status (PS) of 0 or 1; adequate hematologic, hepatic, renal, coagulation, and cardiac function; and no treatment with statins, anticoagulant drugs, or enzyme-inducing products. ECOG PS 0 and 1 were considered equivalent to Karnofsky PS 100 and 80/90, respectively (36). Screening evaluations were conducted within three weeks prior to patient inclusion and consisted of tumor assessment by computed tomography (CT), left ventricular ejection fraction, electrocardiogram, biochemistry and blood count, physical examination, recording ECOG PS, and assessment of concomitant diseases and medications.

Sunitinib was administered at the standard schedule of 50 mg daily for four weeks, followed by a two-week rest period between cycles.

Patients were categorized into favorable, intermediate, and poor risk groups according to the International Metastatic Renal Cell Carcinoma Database Consortium (IMDC) criteria.

Clinical evolution was monitored by CT every other treatment cycle, and tumor measurements were assessed using the Response Evaluation Criteria in Solid Tumors (RECIST) version 1.0.

### 2.3 Sample collection

Blood samples were obtained within 72 hours prior to the start of sunitinib treatment. Samples were collected in tubes without anticoagulants and centrifuged at 1500 x *g* for 30 minutes at 4 °C. Serum was collected, aliquoted, stored at -80 °C, and shipped to a centralized laboratory under appropriate conditions. Blood cell counts and routine biochemistry tests were performed by the local laboratories of the participating centers.

### 2.4 Chemicals and reagents

Acetonitrile and isopropanol (IPA) (LC-MS grade) was obtained from Fisher Scientific (Madrid, Spain), and formic acid (analytical grade) was purchased from Sigma Aldrich Quimica SA (Madrid, Spain). Water of Milli-Q grade was obtained from a Millipore purification system. Standards of various metabolites and internal standards were obtained from Sigma Aldrich Quimica and Toronto Research Chemicals. Standards of 3-indoleacetonitrile (Kioto Encyclopedia of Genes and Genomes (KEGG) code C02938), quinolinic acid (C03722), aminophenol (C01987), 3-hydroxykynurenine (C03227), p-tyrosine (C0082), m-tyrosine (Human Metabolome Data Base ID, HMDB59720), o-tyrosine (HMDB06050), serotonin (C00780), 5-hydroxytryptophan (C00643), L-kynurenine (C00328), phenylalanine (C00079), hydroxyanthranillic acid (C00632), tryptophan (C00078), xanthurenic acid (C02470), tryptamine (C00398), kynurenic acid (C01717), N-acetylserotonin (C00978), phenylacetylglutamine (PAGN) (C04148), indole-3-acetamide (C02693), anthranillic acid (C00108), melatonin (C01598), 3-indoleacetic acid (KEGG C00954), tryptophol (00955), and indolelactic acid (C02043) were obtained from Sigma Aldrich Quimica. N-formylkynurenine (C02700), 6-hydroxymelatonin (C05643), 4-chloro-kynurenine, 5-methoxytryptamine (C05659) and N-formyl-N-acetyl-5-methoxykynurenamine (C05642), and phenylalanine-D_5_ were purchased from Toronto Research Chemicals (Toronto, Canada). The deuterated internal standards melatonin-D_4_, 5-hydroxytryptophan-D_4_, L-kynurenine-D_4_, indole-D_5_-3-acetamide, 4-chloro-kynurenine-^13^C_2_,^15^N, 6-hydroxymelatonin-D_4_, kynurenic acid-D_5_, PAGN-D_5_, phenylalanine-D_5_, serotonin-D_4_, tryptamine-D_4_, tryptophan-D_5_, xanthurenic acid-D_4_, were obtained from Toronto Research Chemicals. Phenylalanine-D_5_ was purchased from Cambridge Isotope Laboratories (Andover, USA). Leukine enkephalin and reserpine were purchased from Sigma-Aldrich Química SA (Madrid, Spain).

### 2.5 Analysis of circulating proteins

Serum proteins were measured using Luminex^®^ panels (Merck Millipore, Burlington, USA) for interleukin (IL)-8, tumor necrosis factor α (TNF-α), and angiopoietin-2 (Ang-2). ELISAs were used to quantify the serum levels of IL-6, VEGF-A, S100 calcium binding A9 (S100A9), tissue factor (TF), and arginase-1 (Arg-1). Each sample was tested in duplicate, and protein concentrations were calculated using linear regression analysis. The results are expressed as pg/mL or ng/mL of protein in serum.

### 2.6 Untargeted lipidomics

Serum samples were thawed on ice. Fifty microliters of serum were withdrawn, and 100 µL of cold acetonitrile (0.1% v/v HCOOH) was added for protein precipitation. The samples were homogenized (vortex, 10 s) and centrifuged at 13000 x g (4 °C, 10 min). The supernatant (120 µL) was collected, transferred to a 96-well plate, and evaporated to dryness under vacuum at 25 °C. 100 µL of cold acetonitrile (0.1% v/v HCOOH) was added for protein precipitation. The samples were then dissolved in a solution of (1:1) (5:1:4 IPA/CH_3_OH/H_2_O, 5 mM CH_3_COONH_4_, 0.1% v/v HCOOH)/(99:1 IPA/H2O, 5 mM CH_3_COONH_4_, 0.1% v/v HCOOH). Blanks were prepared by replacing serum samples with H_2_O:CH_3_OH (1:1, 0.1% v/v HCOOH). A quality control (QC) sample was prepared by mixing 10 µL of each prepared sample in a single glass vial.

The samples were analyzed in a single analytical batch that included 14 QC replicates, 4 blanks, and 75 serum samples. Sample acquisition was randomized, and the QC was analyzed every 7 injections to monitor and correct changes in instrument response. Chromatographic analysis was performed on an Agilent 1290 Infinity UPLC system using a UPLC BEH C18 column (50 x 2.1 mm, 1.7 µm, Waters, Wexford, Ireland). The autosampler and column temperatures were set to 4 °C and 55 °C, respectively, with an injection volume of 2 µL. Binary gradient elution was conducted at a flow rate of 400 µL/min, starting at 98% of mobile phase A (5:1:4 IPA/CH_3_OH/H2O, 5 mM CH_3_COONH_4_, 0.1% v/v HCOOH) for 0.5 min, followed by a linear gradient from 2 to 20% of mobile phase B (99:1 IPA/H_2_O, 5 mM CH_3_COONH_4_, 0.1% v/v HCOOH) for 3.5 min and from 20 to 95% v/v of mobile phase B in 4 min. 95% v/v of mobile phase B was maintained for 1 min, and a return to initial conditions was achieved in 0.25 min and was maintained for a total run time of 14 min. Full scan MS data (60 to 1500 m/z, 6 Hz) were collected on an Agilent 6550 QTOF mass spectrometer in TOF MS mode with electrospray ionization (ESI). The parameters included a gas temperature of 200 °C, drying gas at a flow rate of 14 L/min, a nebulizer pressure at 37 psig, a sheath gas temperature of 350 °C, and a sheath gas flow rate of 11 L/min. Mass reference standards were used for automatic recalibration during analysis.

Peak table generation was performed using XCMS with the centWave method. The following peak detection parameters were used: mass accuracy (15 ppm), peak width (6–20 seconds), and signal-to-noise threshold (50). Peaks were grouped using the nearest method with mzVsRT=1 and tolerances of 9 s and 2 mDa for retention time (RT) and m/z, respectively. The FillPeaks method was applied to fill in missing data. Peak integration and alignment accuracy were verified by comparing automated and manual integration of internal standards, achieving correlation coefficients >0.99. Metabolite identification was performed by matching the m/z values and MS/MS spectra against the Human Metabolome Data Base (HMDB) and METLIN databases with 5 ppm accuracy, using LipiDex (37). Batch effects were corrected using the QC-SVRC and SVR functions in MATLAB (38, 39), excluding features with an RSD(QC) >20%. Blank samples were used to identify and remove contaminants (40). The final dataset included 8285 features (5435 ESI+ and 2789 ESI-).

### 2.7 Targeted metabolic analysis: tryptophan metabolism and amino acid profiling

For the quantification of 29 tryptophan metabolites, phenylalanine, p-tyrosine, m-tyrosine, o-tyrosine, and phenylacetylglutamine, 50 µL aliquots of serum samples were placed into 1.5 mL Eppendorf tubes. Protein precipitation was performed by adding 150 µL of cold acetonitrile. The samples were vortexed for 15 s and centrifuged at 15,000 x g for 10 min at 4 °C. The supernatants were transferred to clean tubes and evaporated in a Thermo SPD121P SpeedVac concentrator (Waltham, MA, USA). The residues were reconstituted in 50 µL of an internal standard solution containing hydroxytryptophan-D_4_, L-kynurenine-D_4_, indole-D_5_-3-acetamide, 4-chloro-kynurenine-^13^C_2_,^15^N, 6-hydroxymelatonin-D_4_, kynurenic acid-D_5_, PAGN-D_5_, phenylalanine-D_5_, serotonin-D_4_, tryptamine-D_4_, tryptophan-D_5_, xanthurenic acid-D_4_, and phenylalanine-D_5_ (900 nM each). The samples were then centrifuged at 15,000 x g for 5 min at 4 °C, and the supernatants were transferred to a 96-well plate for analysis. A diluted sample (dilution factor: 20) was prepared to ensure that metabolites typically present at higher concentrations (e.g., tryptophan) fell within the linear range.

UPLC-MS/MS analysis was performed on an Acquity-Xevo TQS system using MassLynx (Waters) software. An Acquity HSS T3 C18 column (100 x 2.1 mm, 1.8 µm) was used for chromatographic separation. The mobile phases were H_2_O (0.1% v/v HCOOH) (A) and CH_3_CN (0.1% v/v HCOOH) (B). The gradient elution profile was as follows: phase B was held at 2% from 0 to 0.5 min, increased linearly to 45% over 5 min, then to 90% in 0.2 min, followed by a return to initial conditions between 5.7 and 6 min, which were held for 1.5 min for column re-equilibration. The injection volume, flow rate, and column temperature were set at 3 µL, 550 µL/min, and 55 °C, respectively. The autosampler temperature was set at 6 °C during sample analysis. The electrospray ionization conditions were as follows: capillary voltage 2.9 kV, cone voltage 25 V, source temperature 120 °C, desolvation temperature 395 °C, and N_2_ cone and desolvation gas flow rates of 150 and 800 L/h, respectively.

For amino acid analysis, the samples were thawed, vortexed for 15 s, and centrifuged at 10,000 × g for 10 min at 4 °C. Ten µL of each sample was mixed with 60 µL H_2_O. The samples were then derivatized using the AccQTag Ultra (Waters) method: 10 µL of the diluted sample was mixed with 70 µL borate buffer. After vortexing for 15 s, 20 µL of 6-aminoquinolyl-N-hydroxysuccinimidyl carbamate was added. The samples were vortexed for 15 s, incubated at 55 °C for 10 min, and transferred to a 96-well plate for UPLC-MS/MS analysis within 24 hours. Quantitative analysis of amino acids was performed using multiple reaction monitoring (MRM) transitions.

### 2.8 Software and statistical analysis

Categorical variables are reported as frequencies and proportions, and continuous data are reported as medians and ranges. Correlations between experimental findings were calculated using the Pearson correlation method. Univariate and multivariate analyses were conducted to identify predictive factors associated with progression-free survival (PFS) and overall survival (OS) from initial treatment with sunitinib. Initial screening factors were estimated using the Kaplan-Meier method and compared with log-rank tests. Multiple Cox regressions were performed to determine independent predictive factors, including variables that were significant in the univariate analysis (p-value <0.1).

All the statistical analyses were performed using R 4.0.4 (The R Foundation for Statistical Computing, Vienna, Austria). Optimal cutoff values for continuous biomarkers were determined using the cutp function of the survMisc R package (v 0.5.5), which identifies the threshold that maximizes survival differences between groups based on the log-rank statistic. Kaplan-Meier and Cox regressions were performed using the *survfit* and coxph functions from the *survMisc* library (v 3.2-11). Plots were generated using the *survminer* library (v 0.4.9). All tests were two-sided, with a significance level of 5% (α=0.05) and 80% power (β=0.20); p-values <0.05 were considered significant.

Targeted and untargeted UPLC-MS analysis was performed using MassLynx (Waters) and MassHunter (Agilent) software. Peak table generation was performed using XCMS in R. The t-test was used to compare continuous data between two groups. Principal Component Analysis (PCA) and Partial Least Squares-Discriminant Analysis (PLS-DA) were conducted using PLS Toolbox 8.0 (Eigenvector Research Inc., Wenatchee, USA) and in-house MATLAB scripts.

The number of latent variables (LVs) in PLS models was selected using a leave-one-patient-out cross-validation (CV) strategy. The figures of merit included classification accuracy, area under the receiver operating curve (AUROC), sensitivity, selectivity, and negative and positive likelihood ratios. The statistical significance of the CV figures of merit was assessed using permutation testing (n=500). Pathway analysis was conducted with MetaboAnalyst 3.0 (McGill University, Canada), and the Prize-collecting Steiner-forest algorithm for Integrative Analysis of Untargeted Metabolomics (PIUMet) algorithm was used for integrative analysis of untargeted metabolomics.

## 3 Results

### 3.1 Patient characteristics

Forty-five patients were prospectively recruited. Six patients were excluded from the study because blood samples were not available. Therefore, this study included samples collected from 38 patients, including 28 (73.6%) men and 10 (26.3%) women. The median PFS and OS were 9.87 months (95% CI, 5.59 - 14.14) and 21.10 months (95% CI, 11.44 - 30.76), respectively. Patient characteristics and clinical outcomes are summarized in **Table 1** and **Table 2**, respectively. The proportions of patients in the favorable-, intermediate-, and poor-risk IMDC risk groups were 26.3%, 47.3%, and 13.1%, respectively. Five (13.1%) patients could not be classified due to insufficient data. The median OS values for patients in the favorable-, intermediate-, and poor-risk groups were not reached, 15.7 months (95% CI, 12.42 - 18.98), and 8.5 months (95% CI, 8.36 - 8.64), respectively.

**Table 1:** Patient characteristics.

Patient characteristics	Values
Number of patients	38
Gender, N (%)	
Male	28 (73.6)
Female	10 (26.3)
Median age (years)	65.28
ECOG PS, N (%)	
0	7 (18.4)
1	23 (60.5)
NA	8 (21.0)
IMDC risk group (%)	
Favorable	10 (26.3)
Intermediate	18 (47.3)
Poor	5 (13.1)
NA	5 (13.1)
Median time from diagnosis to 1st−line sunitinib, months (range)	12.3 (0.7 - 226.3)
Prior nephrectomy, N (%)	
Yes	30 (78.9)
No	3 (7.9)
NA	5 (13.1)
Metastatic sites, N (%)	
Lymph nodes	23 (60.5)
Lung	27 (71.0)
Bone	9 (23.6)
Liver	3 (10.7)
Others	2 (5.2)
Number of metastatic sites, N (%)	
1	17 (44.7)
2	14 (36.8)
	7 (18.4)
Median duration of sunitinib treatment, months (range)	7.0 (0.3 - 33.5)
Reason for discontinuation, N (%)	
Disease progression	19 (50.0)
Toxicity	4 (10.5)
Clinical deterioration	5 (13.1)
Other	10 (26.3)
Best response to treatment, N (%)	
Complete response	1 (2.6)
Partial response	10 (26.3)
Stable disease	19 (50.0)
Progressive disease	7 (18.4)
NA	1 (2.6)
Median PFS, months (95% CI)	9.87 (5.59 - 14.14)
Median OS, months (95% CI)	21.10 (11.44 - 30.76)

N, number; ECOG PS, Eastern Cooperative Oncology Group performance status; NA, not available; IMDC, International Metastatic Renal Cell Carcinoma Database; PFS, progression-free survival; OS, overall survival; CI, confidence interval.

**Table 2:** Summary the clinical outcomes of the patients included in the analysis, classified according to the considered response phenotypes.

Sample	PFS (months)	OS (months)
1	<=6M	]10:36[
2	NA	]10:36[
3	<=6M	<=10M
4	<=6M	]10:36[
5	]6:22[	>=36M
6	<=6M	<=10M
7	]6:22[	]10:36[
8	]6:22[	]10:36[
9	<=6M	<=10M
10	]6:22[	]10:36[
11	]6:22[	]10:36[
12	<=6M	<=10M
13	<=6M	<=10M
14	]6:22[	]10:36[
15	>=22M	]10:36[
16	]6:22[	]10:36[
17	]6:22[	]10:36[
18	]6:22[	]10:36[
19	NA	]10:36[
20	]6:22[	]10:36[
21	]6:22[	]10:36[
22	>=22M	>=36M
23	]6:22[	]10:36[
24	>=22M	]10:36[
25	<=6M	]10:36[
26	>=22M	]10:36[
27	]6:22[	]10:36[
28	<=6M	]10:36[
29	<=6M	<=10M
30	<=6M	]10:36[
31	<=6M	<=10M
32	<=6M	<=10M
33	>=22M	>=36M
34	]6:22[	]10:36[
35	]6:22[	>=36M
36	<=6M	]10:36[
37	<=6M	<=10M
38	]6:22[	>=36M

### 3.2 Overview of circulating biomarkers

**Figure 1:**
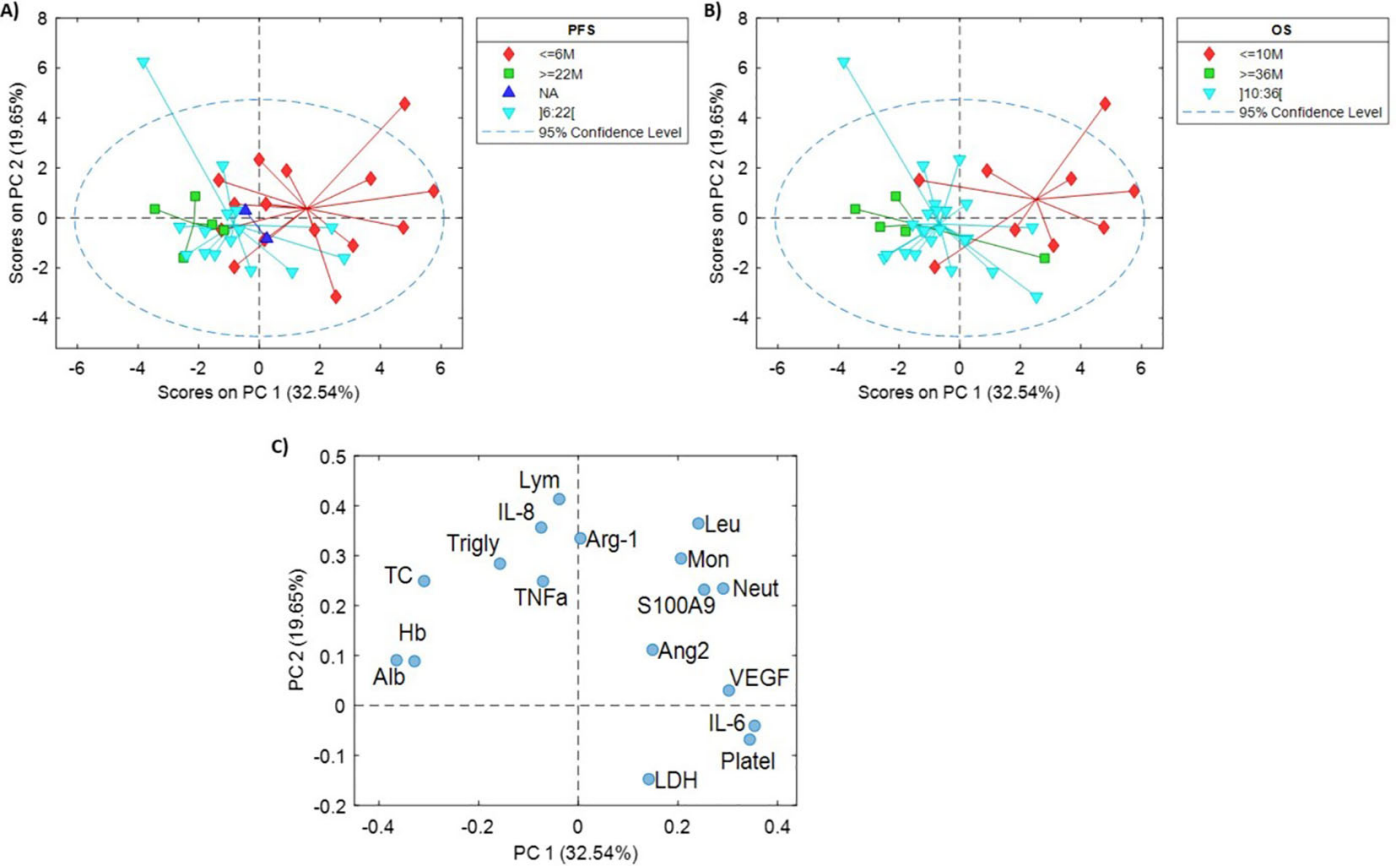
Principal component analysis. PC1 vs PC2 score plots. Samples were labeled according to the PFS **(A)** or OS **(B)** separately for a better visualization. **(C)** Loadings plot from the PCA model. Note: Hb, hemoglobin; Leuc, leukocytes; Neut, Neutrophils; Lym, Lymphocytes; Mon, monocytes; Platel, platelets; Alb, albumin; TC, total cholesterol; Trigly, triglycerides; LDH, lactatodehydrogenase; IL, interleukin; TNFα, tumor necrosis factor-α; VEGF, vascular endothelial growth factor; Ang-2, angiopoietin-2; Arg1, arginase-1; S100A9, S100 Calcium Binding Protein A9.

PCA was employed for an initial explorative analysis of the dataset (**Figure 1**), revealing significant clusters of samples based on extreme discordant phenotypes for PFS (**Figure 1A**) and OS (**Figure 1B**). The analysis identified markers such as hemoglobin (Hb), albumin (Alb), total cholesterol (TC), VEGF, IL-6, leucocytes, monocytes, neutrophils, Ang-2, and S100A9 as potential indicators of clinical outcome. Pairwise correlations between markers further revealed associations between various factors, including hematological parameters and cytokine levels. Joint analysis of the score plots and the loading plot revealed a negative correlation of Hb, Alb, and TC with PFS and OS. Conversely, higher levels of VEGF, IL-6, leucocytes, monocytes, neutrophils, Ang-2, and S100A9 were positively correlated with poor PFS and OS (**Figure 1C**).

**Table 3** summarizes the pairwise correlations between markers. Neutrophil counts showed a strong positive correlation with platelet counts, and with IL-6, VEGF, and S100A9 concentrations. Hb levels were inversely correlated with platelet counts, IL-6, VEGF, and S100A9 concentrations. Alb levels were positively correlated with Hb and TC strongly negatively correlated with neutrophil and platelet counts, and with lactate dehydrogenase (LDH), IL-6, and VEGF concentrations. TC displayed strong positive correlations with Hb and Alb levels, and with lymphocyte counts, as well as negative correlations with platelet counts and the IL-6 and VEGF concentrations. In addition, a strong positive correlation of the IL-6 concentration with platelet counts, and with the LDH and VEGF concentrations was found. The TNF-α concentration significantly correlated with the IL-8 concentration. Positive correlations were also found between Ang-2 concentrations and monocyte counts, and between the Arg-1 and IL-8, and lymphocyte counts. S100A9 levels showed strong correlations with leucocyte counts and with neutrophil counts.

**Table 3:** Pairwise correlations between the concentrations of the selected markers in serum samples.

		Hb	Leu	Neut	Lym	Mon	Platel	Alb	TC	Trigly	LDH	IL-6	TNFα	VEGF	IL-8	Ang2	Arg1
Leucocytes/µL	r	-.132															
	p	.438															
	N	37	**Leu**														
Neutrophils /µL	r	-.216	.939^**^														
	p	.199	.000														
	N	37	37	**Neut**													
Lymphocytes/µL	r	.134	.423^**^	.134													
	p	.430	.009	.430													
	N	37	37	37	**Lym**												
Monocytes /µL	r	-.251	.694^**^	.588^**^	.316												
	p	.133	.000	.000	.057												
	N	37	37	37	37	**Mon**											
Platelets/µL	r	-.688^**^	.350^*^	.467^**^	-.087	.353^*^											
	p	.000	.033	.004	.607	.032											
	N	37	37	37	37	37	**Platel**										
Albumin (g/dL)	r	.773^**^	-.322	-.436^**^	.203	-.327	-.702^**^										
	p	.000	.063	.010	.249	.059	.000										
	N	34	34	34	34	34	34	**Alb**									
TC (mg/dL)	r	.632^**^	-.131	-.348	.478^**^	-.093	-.569^**^	.641^**^									
	p	.000	.497	.064	.009	.633	.001	.000									
	N	29	29	29	29	29	29	29	**TC**								
Triglycerides (mg/dL)	r	.256	.055	-.168	.522^**^	-.050	-.479^**^	.219	.445^*^								
	p	.188	.780	.394	.004	.802	.010	.262	.016								
	N	28	28	28	28	28	28	28	29	**Trigly**							
LDH (mg/dL)	r	-.332	-.069	.011	-.214	.087	.163	-.459^**^	-.431^*^	-.070							
	p	.059	.701	.953	.232	.631	.364	.008	.022	.729							
	N	33	33	33	33	33	33	32	28	27	**LDH**						
IL-6 (pg/mL)	r	-.688^**^	.342^*^	.408^*^	-.106	.302	.626^**^	-.840^**^	-.656^**^	-.200	.529^**^						
	p	.000	.041	.013	.537	.074	.000	.000	.000	.297	.002						
	N	36	36	36	36	36	36	34	30	29	33	**IL-6**					
TNF-α (pg/mL)	r	.228	.160	.113	.177	-.011	-.095	.185	.318	.225	-.163	-.093					
	p	.188	.359	.517	.308	.949	.588	.303	.087	.241	.371	.583					
	N	35	35	35	35	35	35	33	30	29	32	37	**TNFα**				
VEGF (pg/mL)	r	-.526^**^	.327	.405^*^	-.036	.139	.513^**^	-.704^**^	-.519^**^	-.144	.097	.656^**^	-.037				
	p	.001	.051	.014	.837	.418	.001	.000	.003	.458	.592	.000	.829				
	N	36	36	36	36	36	36	34	30	29	33	38	37	**VEGF**			
IL-8 (pg/mL)	r	.056	.061	-.158	.495^**^	.211	-.148	.084	.392^*^	.503^**^	-.151	-.019	.495^**^	.084			
	p	.749	.729	.366	.002	.224	.397	.640	.032	.005	.409	.914	.002	.627			
	N	35	35	35	35	35	35	33	30	29	32	36	36	36	**IL-8**		
Ang-2 (ng/mL)	r	-.215	.254	.246	.082	.429^**^	.108	-.206	-.341	.030	-.072	.196	-.008	.177	.003		
	p	.207	.135	.148	.634	.009	.529	.241	.066	.877	.691	.239	.963	.287	.987		
	N	36	36	36	36	36	36	34	30	29	33	38	37	38	36	**Ang2**	
Arg-1 (pg/mL)	r	-.110	.295	.118	.477^**^	.193	-.105	.047	.222	.338	-.146	-.002	.207	.123	.566^**^	-.028	
	P	.542	.096	.514	.005	.281	.563	.801	.246	.079	.443	.992	.226	.482	.000	.871	
	N	33	33	33	33	33	33	31	29	28	30	35	36	35	34	35	**Arg1**
S100A9 (ng/mL)	r	-.398^*^	.657^**^	.701^**^	.171	.386^*^	.327	-.370^*^	-.261	-.042	-.141	.312	.075	.357^*^	-.017	.262	.272
	p	.022	.000	.000	.341	.026	.063	.040	.171	.831	.459	.068	.670	.036	.926	.129	.115
	N	33	33	33	33	33	33	31	29	28	30	35	35	35	34	35	35

Hb, hemoglobin; Leuc, leukocytes; Neut, Neutrophils; Lym, Lymphocytes; Mon, monocytes; Platel, platelets; Alb, albumin; TC, total cholesterol; Trigly, triglycerides; LDH, lactatodehydrogenase; IL, interleukin; TNFα, tumor necrosis factor-α; VEGF, vascular endothelial growth factor; Ang2, angiopoietin-2; Arg1, arginase-1; S100A9, S100 Calcium Binding Protein A9; r, Pearson correlation coefficient; p, p-value; N, number; *p <0.05; **p <0.01.

### 3.3 Analysis of circulating proteins

The associations between protein levels and survival outcomes were assessed. Univariate analysis revealed that IL-6, VEGF-A, Arg-1, and S100A9 levels were correlated with worse PFS (**Table 4**) and OS (**Table 5**). According to the multivariate analysis, Arg-1 and S100A9 maintained statistical significance for both PFS and OS. Furthermore, the combination of IL-6 and S100A9 demonstrated enhanced prognostic value, with patients exhibiting high levels of both proteins showing significantly worse OS (**Table 5**).

**Table 4:** Univariate and multivariate analysis of proteins for progression-free survival (PFS).

			Univariate analysis			Multivariate analysis	
Parameter	Cut-off value	N	Median PFS, months (95% CI)	HR (95% CI)	*p*-value (≤0.1)	HR (95% CI)	*p*-value
IL-6 (pg/mL)	≤3.87	14	23.9 (9.87 - 45.8)	3.1 (1.39 - 6.92)	0.006	1.19 (0.38 - 3.77)	0.801
	>3.87	23	6.03 (4.73 - 13.9)				
IL-8 (pg/mL)	≤22.5	23	11.3 (5.5 - 17.1)	1.8 (0.86 - 4.04)	0.10	1.49 (0.64 - 3.50)	0.355
	>22.5	12	6.0 (0.0 - 13.5)				
VEGF-A (pg/mL)	≤340	19	23.8 (6.2 - 41.3)	2.2 (1.06 - 4.61)	0.034	1.54 (0.55 - 4.26)	0.263
	>340	19	6.0 (0.0 - 12.7)				
Ang-2 (ng/mL)	≤2	19	14.7 (8.5 - 20.9)	1.79 (0.88 - 3.6)	0.107	2.1 (0.92 - 4.6)	0.081
	>2	18	5.1 (3.3 - 6.8)				
Arg-1 (pg/mL)	≤7600	28	13.2 (9.2 - 24.0)	3.4 (1.39 - 8.38)	0.007	3.2 (1.05 - 10.0)	0.041
	>7600	7	4.0 (2.33 - NA)				
S100A9 (ng/mL)	≤43.08	29	13.9 (9.5 - 24.0)	4.9 (1.8 - 13.7)	0.002	4.3 (1.23 - 15.02)	0.022
	>43.08	6	3.8 (2.6 - NA)				

N, number; PFS, progression-free survival; CI, confidence interval; HR, hazard ratio; IL, interleukin; VEGF, vascular endothelial growth factor; Ang-2, angiopoietin-2; Arg-1, arginase-1; S100A9, S100 Calcium Binding Protein A9.

**Table 5:** Univariate and multivariate analysis of proteins for overall survival.

			Univariate analysis			Multivariate analysis	
Parameter	Cut-off value	N	Median OS, months (95% CI)	HR (95% CI)	*p*-value (≤0.1)	HR (95% CI)	*p*-value
IL-6 (pg/mL)	≤3.87	15	NR (33.7 - NA)	6.0 (2.0 - 18.0)	0.001	3.2 (1.04 - 10.0)	0.043
	>3.87	23	13.2 (9.97 - 24.3)				
IL-8 (pg/mL)	≤22.5	24	33.4 (19.3 - 47.4)	2.1 (0.88 - 5.03)	0.096	1.08 (0.36 - 3.27)	0.549
	>22.5	12	13.03 (4.1 - 21.9)				
VEGF-A (pg/mL)	≤340	19	33.7 (16.6 - 50.9)	2.5 (1.06 - 5.7)	0.037	1.39 (0.35 - 5.56)	0.609
	>340	19	13.2 (5.7 - 20.7)				
Ang-2 (ng/mL)	≤2	19	23.0 (1.1 - 44.9)	1.9 (0.85 - 4.45)	0.10	1.79 (0.60 - 5.35)	0.173
	>2	19	13.2 (9.3 - 17.2)				
TF (pg/mL)	≤625	25	28.6 (15.7 - 43.2)	2.1 (0.88 - 4.98)	0.089	1.01 (0.27 - 3.67)	0.762
	>625	12	15.3 (9.97 - 20.9)				
Arg-1 (pg/mL)	≤7600	28	33.4 (15.7 - NA)	3.2 (1.25 - 8.26)	0.015	2.9 (1.01 - 8.65)	0.047
	>7600	7	13.0 (8.43 - NA)				
S100A9 (ng/mL)	≤43.08	29	28.6 (15.9 - NA)	5.8 (2.04 - 16.7)	0.001	5.9 (1.5 - 22.7)	0.011
	>43.08	6	9.2 (8.4 - NA)				

N, number; OS, overall survival; CI, confidence interval; HR, hazard ratio; IL, interleukin; NR, not reached; NA, not available; VEGF, vascular endothelial growth factor; Ang-2, angiopoietin-2; TF, tissue factor; Arg-1, arginase-1; S100A9, S100 Calcium Binding Protein A9.

**Figure 2:**
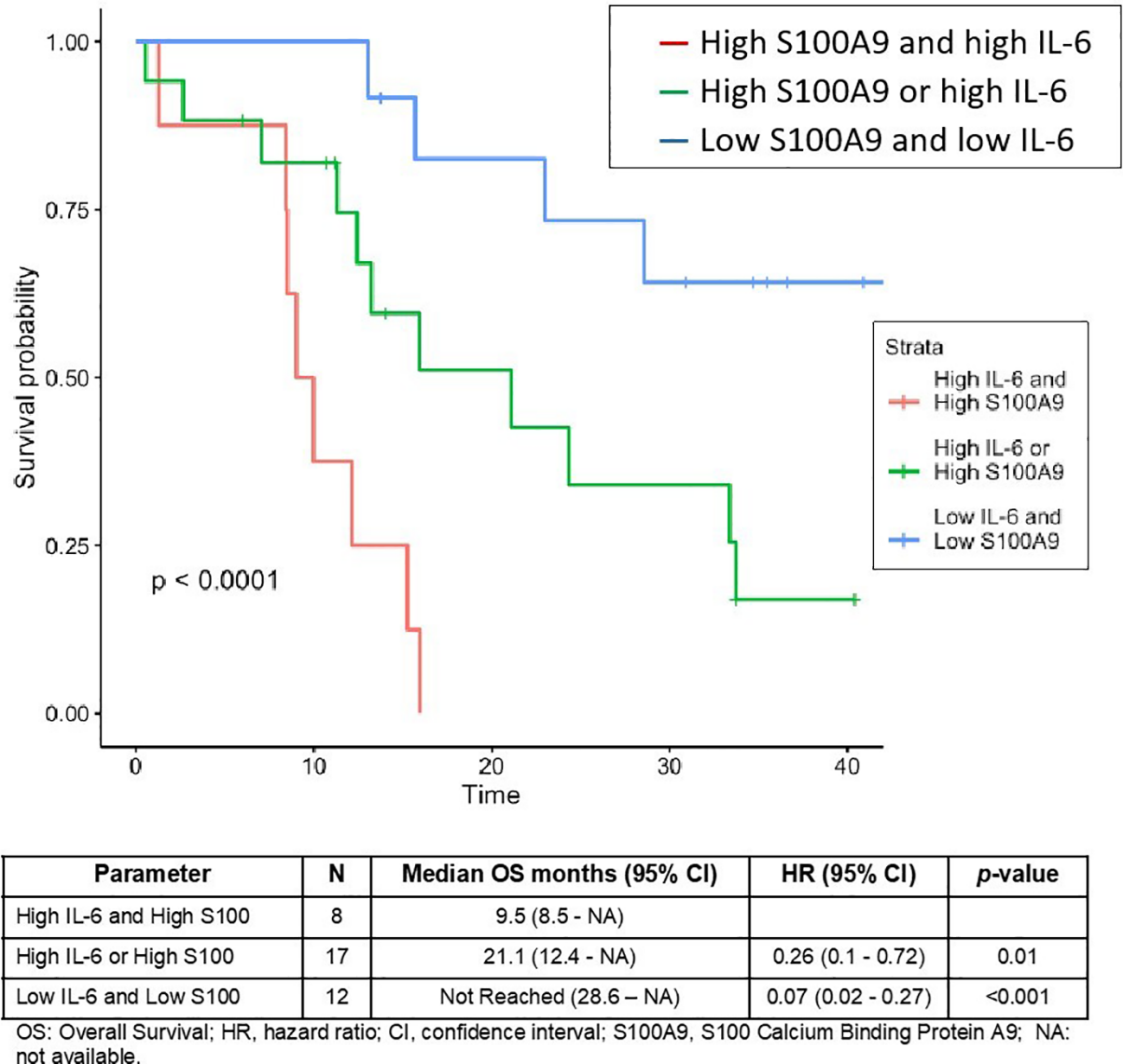
Survival functions for combined levels of circulating proteins S100A9 and IL-6.

We next explored the value of combining IL-6 and S100A9, the two proteins with greatest impact on OS, via multivariate analysis. Patients with high levels of both proteins had worse OS (9.5 months) than patients with either the protein alone (21.1 months, p-value = 0.01), and worse OS than patients with low concentrations of both proteins (p-value <0.001) (**Figure 2**). Kaplan-Meier curves were used to identify 3 risk groups based on high/low levels of S100A9 and IL-6. Based on Arg-1 secretion by MDSCs (31), we focused on circulating Arg-1 levels and IL-6 plus S100A9 and found a 35.6% difference in Arg-1 levels between the high and low groups. Arg-1 levels increased 60% between patients with low and high IL-6 plus IL-8 levels. However, these differences were not statistically significant.

### 3.4 Overview of metabolic profiles

**Figure 3:**
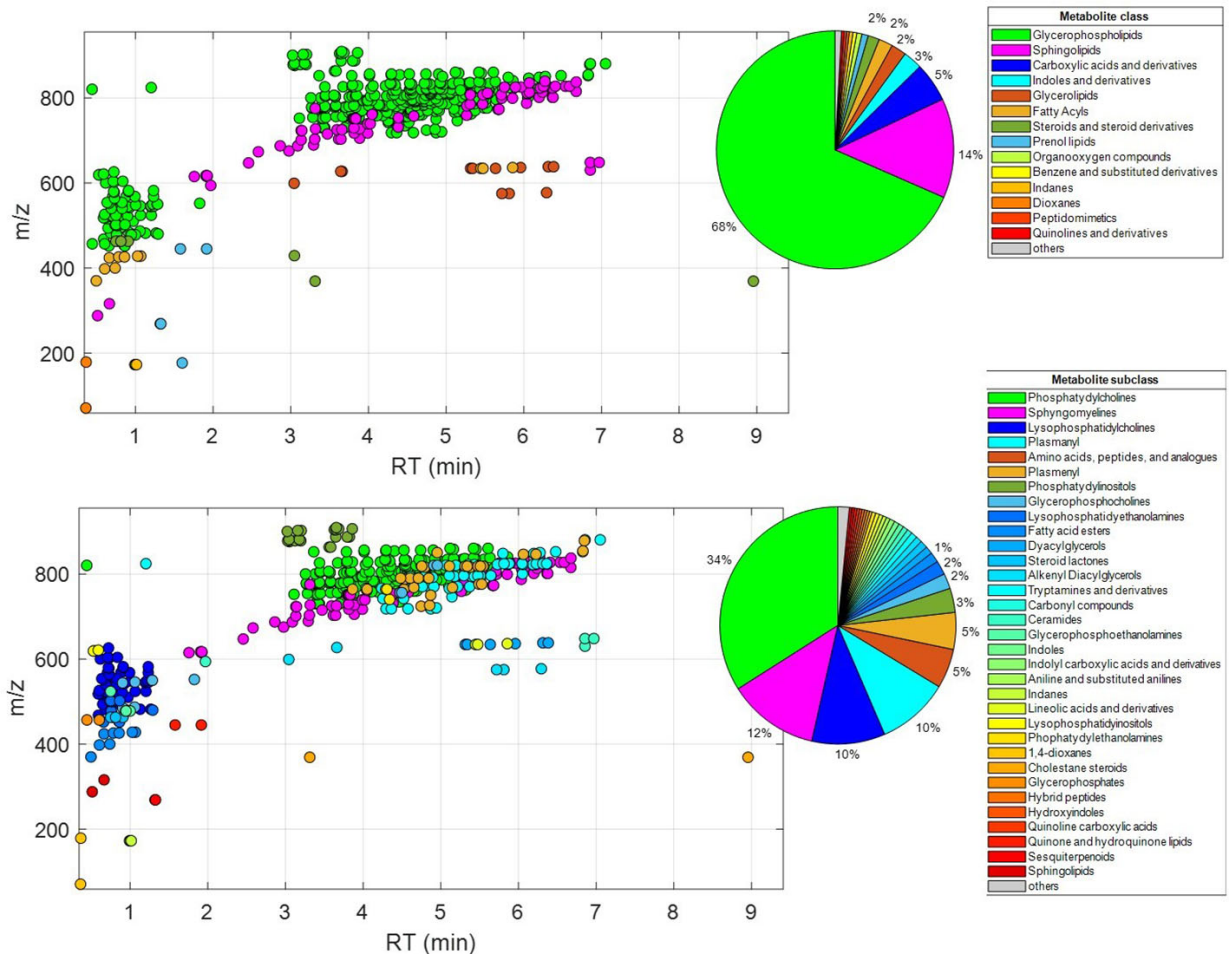
Distributions of lipids annotated based on MS/MS data labeled according to their metabolic class (top) or subclass (bottom).

**Figure 4:**
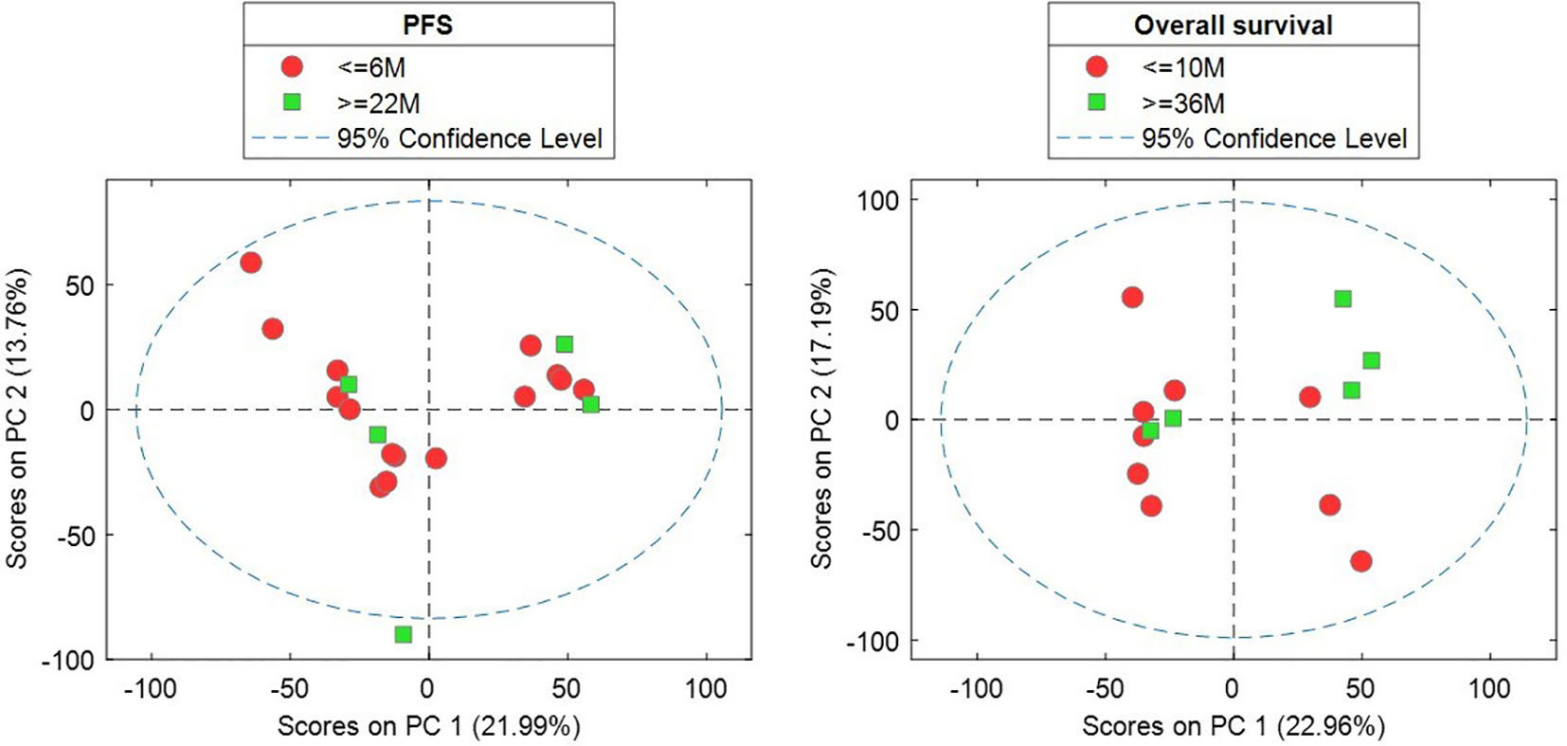
Scores plots of two-component PCA models of the sample subsets used for the analysis of the association between the metabolic profile before treatment and type of extreme response phenotypes based on PFS or OS.

A comprehensive analytical strategy integrating targeted and untargeted approaches was employed to profile the serum metabolome. A wide array of metabolites, particularly glycerophospholipids and sphingolipids, were annotated based on mass spectrometry data (**Figure 3**). PCA revealed overlapping trends in metabolic profiles between different response phenotypes, indicating complex interrelationships (**Figure 4**). Two-component PCA models were plotted (**Figure 4**) to show the association between the metabolic profile before treatment and the type of extreme response phenotypes based on PFS and OS. No sample was classified as a potential outlier based on its Hotelling’s T^2^ statistic at the 95% confidence level. PCA scores showed a high overlap between the classes in the two models, and projections in later PCs did not provide additional sample clustering.

### 3.5 Association between extreme PFS phenotypes and metabolic profiles

**Figure 5:**
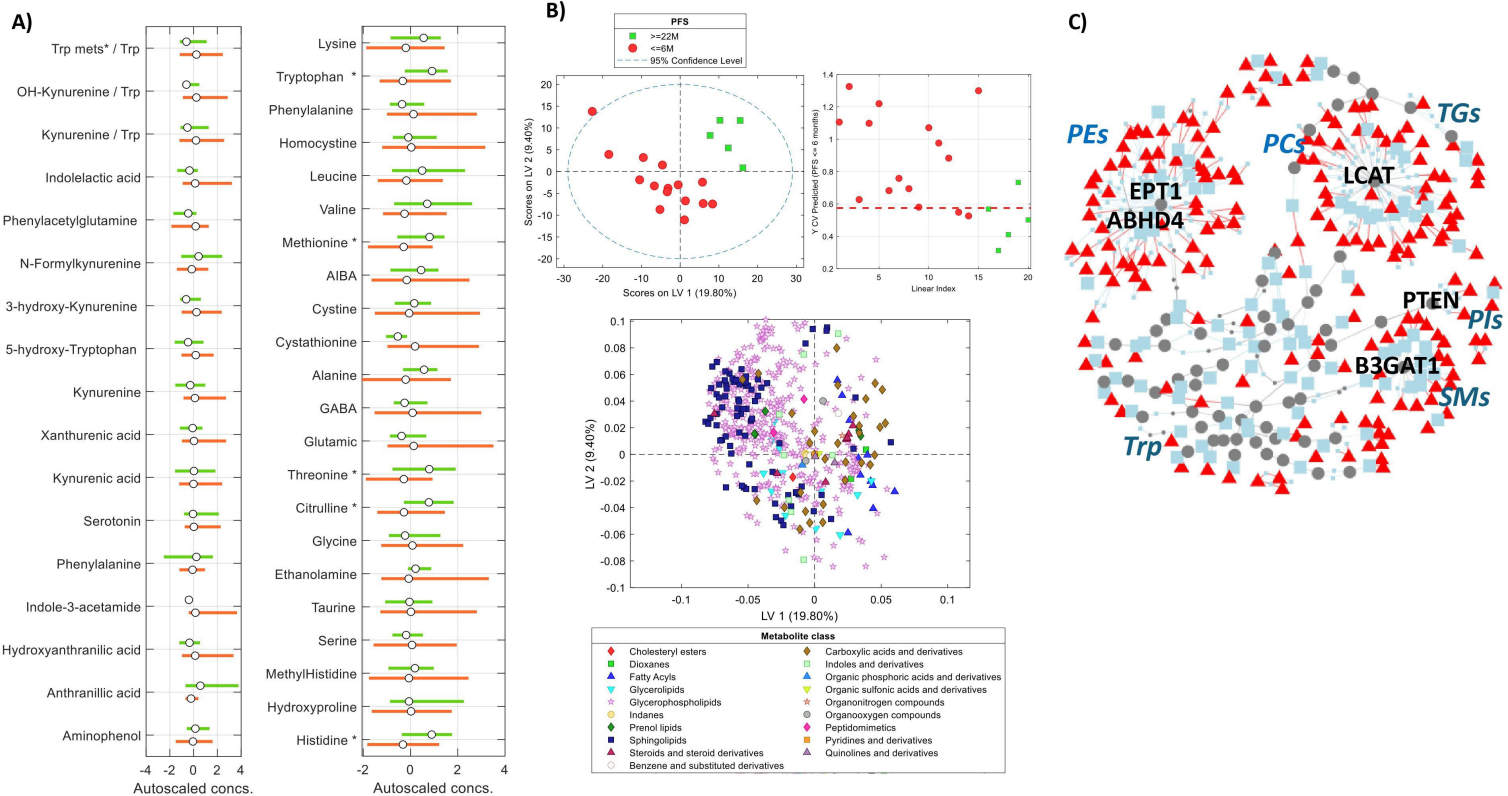
Analysis of the association between PFS extreme phenotypes and the metabolic profiles. **(A)** Boxplots show the distributions of concentrations of Trp and Kyn metabolites and AAs in the two considered groups of patients. Green bar: PFS≥22 months, orange bar, PFS ≤ 6 months; **(B)** PLSDA analysis. Left) PLS1 vs PLS2 scores from the PLS-DA; Right) results from model cross validation; Bottom) PLS loading plots showing the association among metabolic features in the multivariate model; **(C)** Network of protein-protein and protein-metabolite interactions inferred by PIUMet. Types of nodes: Blue squares: hidden metabolites; Gray circles: hidden proteins; Red triangles: metabolite features.

Multivariate discriminant models successfully differentiated between extreme PFS phenotypes (≤6 months vs. ≥22 months), with significant clustering observed in the PLS-DA score plots. Univariate analysis showed that several discriminant metabolites, particularly glycerophospholipids and sphingolipids, were associated with PFS (**Figure 5**). To assess whether the tryptophan metabolism pathway was significantly associated with these extreme PFS phenotypes, pathway analysis was conducted using autoscaled data. Enrichment analysis was performed using the global test, and pathway topology was evaluated based on relative betweenness centrality, with reference to the KEGG *Homo sapiens* database. This analysis did not reveal a statistically significant association between extreme PFS phenotypes and the tryptophan metabolism pathway. To extract more information from the observed differences in the metabolic profiles, the PIUMet algorithm was used for the analysis of associations among the set of features classified as discriminant (t-test p-value<0.05).

### 3.6 Association between OS and metabolic profile

**Figure 6:**
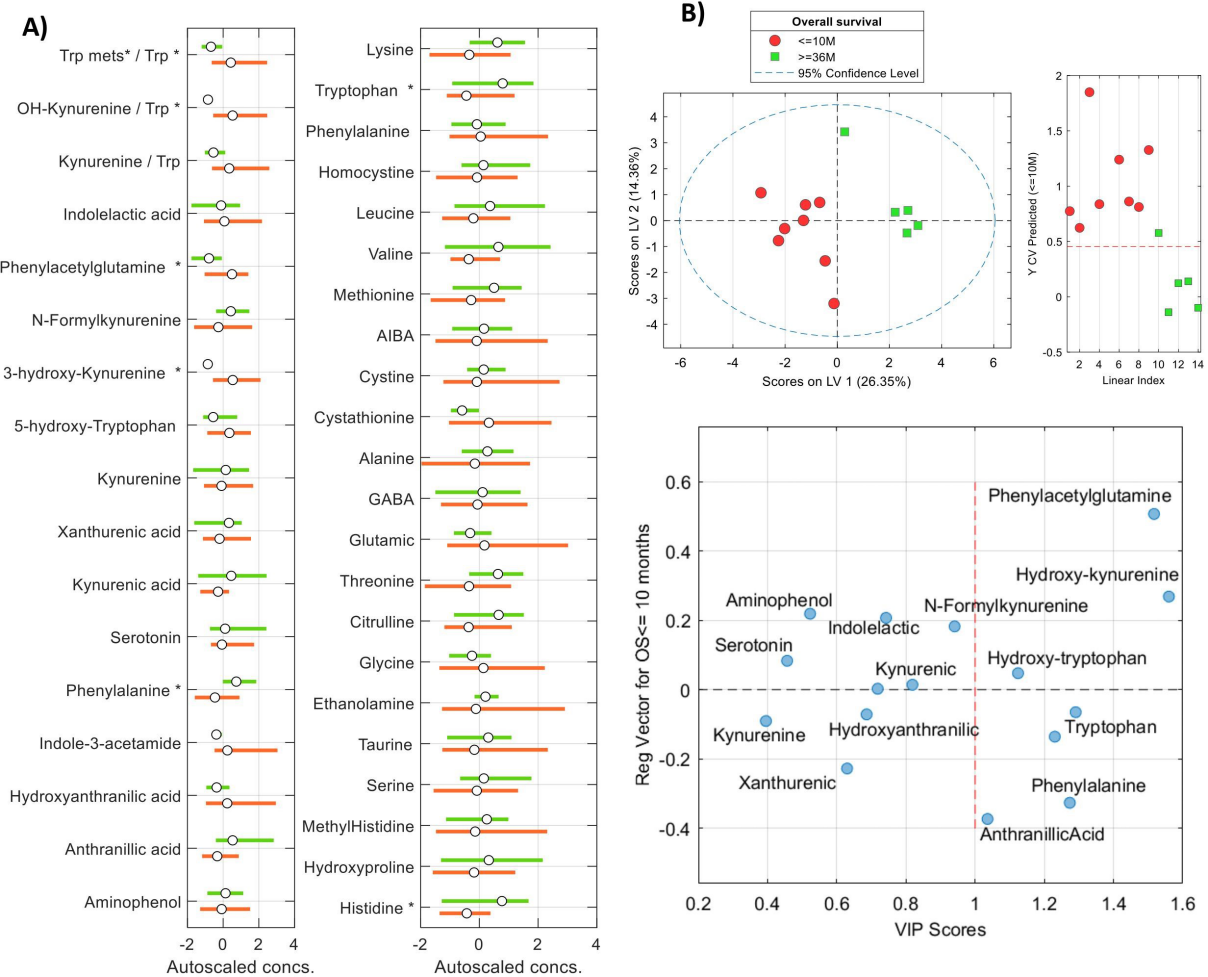
Analysis of the association between OS extreme phenotypes and the metabolic profiles. **(A)** Boxplots showing the distributions of concentrations of Trp and Kyn metabolites in the two considered groups of patients; **(B)** PLSDA analysis using a set of 16 features involved in tryptophan metabolism. Top left) PLS1 vs PLS2 scores from the PLS-DA model; Top right) results from model cross validation; Bottom) PLS regression vector showing the association among metabolites in the multivariate model and the VIP score.

**Figure 7:**
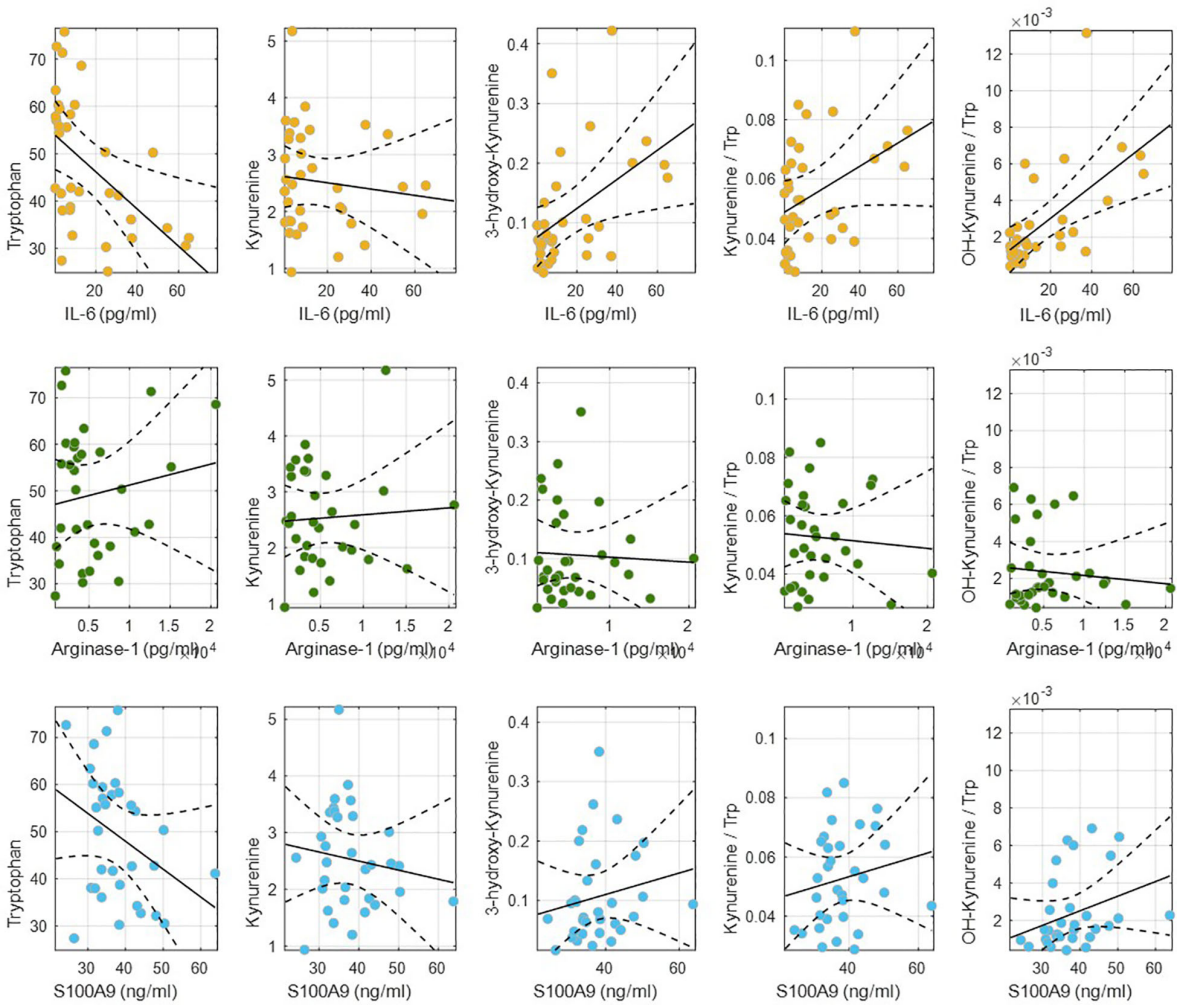
Linear models showing the relationship between Tryptophan (Trp), Kynurenine (Kyn), hydroxykynurenine (OHKyn) concentrations, and the Trp/Kyn and OHKyn/Trp ratios with IL-6, Arg-1, and S100A9.

A similar analysis was conducted to assess the association between extreme OS phenotypes (≤10 months vs. ≥36 months) and metabolic profiles. Univariate analysis highlighted alterations in amino acid and tryptophan metabolism, while PLS-DA confirmed significant differences between the two OS groups. Univariate analysis to assess the correlation between the metabolic concentrations and the OS extremes showed altered amino acids (histidine, tryptophan, phenylalanine), tryptophan pathway metabolites (hydroxykynurenine, hydroxytryptophan, and other metabolites (phenylacetylglutamine) (**Figure 6**). Linear models showing the relationship between tryptophan, kynurenine, hydroxykynurenine concentrations, and the Trp/Kyn and OHKyn/Trp ratios with IL-6, Arg-1, and S100A9 are depicted in **Figure 7**. While linear associations were found between the IL-6 levels and the Trp, OHKyn concentrations, as well as with the Kyn/Trp and OHKyn/Trp ratios, no linear associations were found between these metabolites and the levels of Arg-1 and S100A9 (**Figure 6**, **Table 6**). Using the approach described above, pathway analysis further demonstrated a statistically significant association between extreme OS phenotypes and the tryptophan metabolism pathway (p = 0.016).

**Table 6:** Linear models showing the relationship between Tryptophan (Trp), Kynurenine (Kyn), hydroxykynurenine (OHKyn) concentrations, and the Trp/Kyn and OHKyn/Trp ratios with IL-6, Arg-1, and S100A9, including slope, R², and p-value.

	IL-6			Arg-1			S100A9		
	Slope	R^2^	p-value	Slope	R^2^	p-value	Slope	R^2^	p-value
Trp	-0.39	0.26	1 10^-3^	5 10^-4^	0.02	0.4	-0.59	0.12	0.05
Kyn	0.00	0.01	0.5	1 10^-5^	4 10^-3^	0.7	-0.02	0.02	0.4
OHKyn	2 10^-3^	0.24	2 10^-3^	-8 10^-7^	2 10^-3^	0.8	1 10^-3^	0.03	0.3
Kyn/Trp	4 10^-4^	0.15	0.02	-2 10^-7^	6 10^-3^	0.7	-2 10^-7^	6 10^-3^	0.7
OHKyn/Trp	9 10^-5^	0.40	5 10^-5^	-5 10^-7^	0.01	0.6	7 10^-5^	0.10	0.07

These findings underscore the intricate relationship between circulating biomarkers, metabolic profiles, and clinical outcomes in patients with metastatic renal cell carcinoma, providing valuable insights for prognostication and therapeutic decision-making.

## 4 Discussion

In this work, we evaluated the prognostic implications of circulating proteins related to angiogenesis and tumor immune escape in patients with metastatic clear cell renal cell carcinoma (CCRCC) treated with first-line sunitinib. Histological grading has been widely used for decades as a prognostic factor. As new therapies become available, many efforts have been made to identify prognostic factors or predictors of treatment efficacy in patients with RCC. On one hand, genomic and transcriptomic biomarkers have been found to be predictors of the efficacy of sunitinib and other TKIs (41–46). On the other hand, different prognostic models have been built based on clinical and blood test parameters, which are readily available in clinical practice (47–53). Concentrations of circulating proteins provide information on the characteristics of tumors and their microenvironment and can be easily determined before and throughout treatment at a relatively low cost. Consistent with the characteristics of RCC, most of the proteins studied are related to the immune system and angiogenesis, the so-called cytokine and angiogenic factors (CAFs).

Among the CAFs included in the present study, VEGF-A and Ang-2 are directly related to angiogenesis. VEGF-A showed prognostic value for both PFS and OS in the univariate analysis but lost statistical significance when analyzed jointly with the whole set of proteins. Ang-2, a ligand of the Tie-2 receptor along with Ang-1, confers resistance to anti-VEGF therapy and, in conjunction with VEGF, promotes neovascularization and plays a role in immunosuppression through stimulation of cytotoxic T lymphocyte antigen 4 (CTLA-4) and Programmed Death Ligand 1 (PD-L1) (54). Plasma levels of Ang-2 are associated with increased tumor size and high grade (55). Baseline serum samples from 74 patients with advanced RCC treated with sunitinib were analyzed using two multiplex platforms; lower Ang-2 and higher matrix metalloproteinase-2 (MMP2) were associated with tumor response. No information on tumor subtypes is available (56). In the present study, univariate and multivariate analyses showed no impact of Ang-2 on PFS or OS. Tie-2-expressing circulating monocytes are attracted to the tumor microenvironment by endothelial cell-derived Ang-2 (57). Interestingly, we found a strong correlation between serum Ang-2 levels and peripheral blood monocyte counts.

IL-6 is a pleiotropic proinflammatory cytokine that is mainly secreted by monocytes and macrophages, and by different tumor cells (58). Serum IL-6 levels are elevated in patients with RCC, and are correlated with tumor stage and grade (59). A meta-analysis of nearly two thousand RCC patients concluded that high serum or tumor IL-6 levels were associated with shorter survival (60). After treatment with sunitinib and other TKIs, RCC cells secrete IL-6, which induces drug resistance (61). In melanoma patients, IL-6 and IL-8 plasma levels were used jointly to define three prognostic groups (62). Serum IL-6 levels had previously been associated with reduced survival and treatment resistance in melanoma patients, while serum IL-8 concentrations correlated with tumor burden and prognosis in patients with different tumor types, including melanoma and RCC (reviewed in (63)). Moreover, IL8 mediates resistance to sunitinib in RCC cells (64).

Beyond their prognostic value, these biomarkers may also be biologically linked to resistance to anti-angiogenic therapies such as sunitinib. IL-6 activates multiple survival pathways, including JAK/STAT3, PI3K/AKT/mTOR, and HIF signaling, all of which can promote tumor cell survival, angiogenesis, and resistance to VEGF-targeted therapies. In addition, S100A9-driven myeloid inflammation may contribute to a pro-tumorigenic microenvironment that reduces treatment efficacy. Therefore, elevated IL-6 and S100A9 levels may not only reflect poor prognosis but may also represent biological mechanisms associated with resistance to anti-angiogenic therapy.

In patients with metastatic RCC included in a randomized trial of pazopanib vs. placebo (65), pretreatment plasma concentrations of IL-6 and IL-8, among other proteins, were measured. High concentrations of IL-8 predicted shorter PFS in patients treated with pazopanib, high concentrations of IL-6 or IL-8 predicted shorter PFS in the placebo group, and high concentrations of IL-6 were predictive of improved relative PFS benefit from pazopanib. Interestingly, these markers were stronger predictive factors than the standard risk classifications by the Memorial Sloan Kettering Cancer Center (49) and the IMDC (48).

Elevated tumor S100A9 expression has been associated with shorter PFS and cancer-specific survival in patients with CCRCC who underwent nephrectomy (66). In the present study, multivariate analysis showed that the IL-6, Arg-1, and S100A9 levels predicted OS, while the S100A9 and Arg-1 levels were predictive of both PFS and OS. More interestingly, combined levels of IL-6 and S100A9 (both proteins high, either one of the two proteins high or both proteins low) identified three significantly different risk patient groups, although a larger number of patients would be required to establish its clinical value. In any case, the strong association of low levels of both proteins with longer survival (p<0.0001) is remarkable. Given the relationships of IL-6 and S100A9 with the antitumor capacities of the immune system, it would be interesting to evaluate the proposed composite marker in patients treated with either TKIs, immunotherapy, or combined treatments. Moreover, as mentioned above, S100A9 is involved in several protumor mechanisms, in addition to the accumulation and activation of MDSCs. Nonetheless, our results should be interpreted with caution due to the small number of patients in each group.

Our results suggest that IL-6, S100A9, and Arg-1 should not be interpreted as independent biomarkers but rather as components of a common biological process related to systemic inflammation, myeloid cell activation, and tumor immune escape. IL-6 is a central inflammatory cytokine that activates the JAK/STAT3 pathway and promotes the expression of IDO1, leading to increased tryptophan catabolism and kynurenine production. Kynurenine and other tryptophan metabolites suppress T-cell function and promote regulatory T cells, contributing to immune escape. In parallel, S100A9 promotes the accumulation and activation of myeloid-derived suppressor cells, which produce Arg-1 and further inhibit T-cell proliferation through arginine depletion. Therefore, IL-6, tryptophan metabolism, S100A9, and Arg-1 may represent different components of a shared immunometabolic axis associated with tumor progression and poor prognosis in CCRCC.

As expected, many correlations were found between different markers. Total cholesterol (TC) levels displayed strong positive correlations with Alb and Hb levels and with lymphocyte counts, and negative correlations with platelet counts and with IL-6 and VEGF concentrations (**Table 3**). These findings are consistent with studies that associate high cholesterol levels with better clinical outcomes in RCC (67, 68), and suggest that cholesterol is a candidate for inclusion in multiparametric prognostic models.

Tryptophan metabolism has been implicated in various aspects of cancer biology (8), including tumor growth, immune evasion, and metastasis. In RCC specifically, tryptophan metabolism is enhanced, and indoleamine 2,3-dioxygenase (IDO) expression is increased in both tumor cells and in the microenvironment of human RCC compared to normal kidney tissue (69). IDO-1 is an intracellular and immunosuppressive rate-limiting enzyme in the metabolism of tryptophan to kynurenine with immunosuppressive effects. Tryptophan concentration in the cell microenvironment is reduced by its metabolization, which causes T-cell arrest in the G1 phase of the cell cycle, inhibiting T-cell proliferation. Furthermore, kynurenine induces T cell apoptosis, and promotes the balanced differentiation of Th17/Treg to Treg cells (6, 70). Recent findings suggest that in metastatic RCC, IDO-1 may be a more promising predictive biomarker for response to immune-based cancer therapy (Nivolumab) than PD-L1 (71). The results depicted in **Figure 5A** from the targeted analysis of the tryptophan pathway showed that the PFS ≤ 6 group of samples was characterized by higher concentrations of tryptophan-metabolites (e.g., kynurenine, 3-hydroxykynurenine, hydroxyanthranilic acid, ratio 3-hydroxykynurenine/tryptophan) and lower levels of tryptophan, in agreement with previous results found in RCC (12). As mentioned above, tryptophan metabolism has been identified as a critical factor in progressive cancer (8), and it has been previously reported that many cancers upregulate a liver enzyme, tryptophan dioxygenase, to drive tryptophan consumption. Furthermore, a primary product of this process, kynurenine, is an endogenous ligand for the aryl hydrocarbon receptor, which mediates invasive tumor growth (72). Previous results have demonstrated the involvement of the kynurenine pathway enzymes and catabolites in CCRCC via both immune and non-immune mechanisms (12). These results are in agreement with reports that associated the upregulation of IDO in cancer patients with poor prognosis (73), and with results suggesting the kynurenine to tryptophan ratio could serve as a marker of CCRCC aggressiveness and a prognostic factor for cancer-specific survival and PFS (12). Despite the limited sample size, our results suggest that a more comprehensive profiling of the tryptophan pathway, including kynurenine metabolites, may provide a more accurate identification of alterations in the tryptophan pathway than the use of a single ratio such as kynurenine/tryptophan.

The results depicted in **Figure 5A** show higher concentrations of methionine, threonine, citrulline, and histidine in the PFS ≤ 6 group. Citrulline is a non-essential amino acid involved in several metabolic pathways with important implications for immune function, including arginine production. Citrulline is a major metabolite of glutamine, which is known to support cancer cell growth by fuelling mitochondrial biogenesis and nucleotide biosynthesis. Moreover, high levels of citrulline have been associated with immunosuppression by enhancing the proliferation of MDSCs in cancer patients. On the other hand, low levels of glutamine and histidine, which are conditionally essential amino acids, might reflect their high consumption by rapidly proliferating cancer cells to sustain growth. For instance, glutamine is a critical source of carbon and nitrogen for cancer cells and is required for the synthesis of nucleotides, proteins, and lipids. Furthermore, histidine is converted into histamine, which can promote angiogenesis and modulate immune responses, favoring tumor growth (74).

The analysis of the PLSDA loading plot, as illustrated in **Figure 5C**, enabled the identification of lipid classes associated with the two considered PFS phenotypes. Higher levels of several fatty acyls such as acylcarnitines, carboxylic acids, and derivatives including citrulline, histidine, and methionine were positively associated with PFS≥22. Conversely, sphingolipids such as sphingomyelins and glycerolipids such as diglycerides and alkenyl-diglycerides were associated with the PFS ≤ 6 group.

The network of protein−protein and protein−metabolite interactions inferred by PIUMet, depicted in **Figure 5C**, included 56 hidden proteins, among which were Lecithin-Cholesterol Acyltransferase (LCAT) and Ethanolamine Phosphotransferase 1 (EPT1). LCAT primarily participates in lipid metabolism, especially in the metabolism of high-density lipoprotein (HDL) particles. Changes in cholesterol metabolism might impact the lipid composition of cell membranes, thereby affecting cell signaling pathways and interactions with the tumor microenvironment (75) and in antigen-presenting function (76). Additionally, the accumulation of cholesterol in natural killer cells activates their effector functions against hepatoma cells. Serum TC and low-density cholesterol levels have been found to correlate with the cholesterol content of peripheral NK cells in healthy humans, while in hepatocellular carcinoma patients, these levels correlate with the number and toxicity of intratumor NK cells (77). EPT1 is an enzyme involved in phospholipid biosynthesis, specifically in the synthesis of phosphatidylethanolamine (PE), a major component of cell membranes. PE plays a crucial role in maintaining cell membrane integrity, fluidity, and function (78).

Analysis of the metabolic profiles of patients with extreme OS phenotypes revealed that altered concentrations of certain amino acids and metabolites, particularly those related to tryptophan metabolism, were associated with extreme OS. A PLS-DA model confirmed specific metabolites showing notable importance, including tryptophan, hydroxykynurenine, hydroxytryptophan, and phenylacetylglutamine among the key metabolites identified. Furthermore, results showed statistically significant associations between the IL-6 levels and the Trp and OHKyn concentrations, as well as with the Kyn/Trp and OHKyn/Trp ratios. Chronic inflammation, driven by cytokines that regulate the tumor microenvironment, is recognized as a hallmark of cancer. IL-6, a key cytokine linking inflammation and cancer, modulates a complex array of processes. Notably, kynurenine, produced from tryptophan by the action of IDO, activates the aryl hydrocarbon receptor (AhR), which induces the synthesis of IL-6. IL-6, in turn, induces IDO expression by activating STAT3, leading to an AhR–IL-6-STAT3 signaling loop (79).

In the current therapeutic landscape of metastatic CCRCC, where immune checkpoint inhibitors alone or in combination with TKIs represent the standard first-line treatment, the clinical relevance of these biomarkers may extend beyond patients treated with sunitinib. Both IL-6 and S100A9 are closely associated with the development of an immunosuppressive tumor microenvironment, particularly through the expansion and activation of myeloid-derived suppressor cells and chronic inflammatory signaling. This biological context has been associated with reduced efficacy of immune checkpoint inhibitors. Therefore, elevated circulating IL-6 and/or S100A9 levels may identify patients with a myeloid-driven immunosuppressive phenotype potentially less responsive to immune checkpoints inhibitors (ICI) monotherapy and more suitable for combination strategies such as ICI plus TKI.

Our study demonstrates a prognostic role for IL-6, S100A9, and Arg-1, as these biomarkers are associated with survival outcomes. Due to the single-arm design of the study, we cannot formally establish its predictive value. However, given that IL-6 and S100A9 are involved in TKI resistance and tumor immunosuppression, these biomarkers could have predictive value in patients undergoing IO and TKI treatments These hypotheses should be evaluated in prospective clinical studies.

In summary, this study established the prognostic value of a set of circulating proteins in patients with CCRCC treated with sunitinib. Correlations of these proteins with each other and with different parameters of routine blood tests were determined, and a marker based on the combined levels of two proteins that could distinguish three risk patient groups was identified, although its usefulness should be confirmed with a larger number of patients. Moreover, the study distinguished metabolic patterns related to clinical outcomes, especially those related to the tryptophan-kynurenine pathway.

From a clinical perspective, these biomarkers could be incorporated into future therapeutic strategies at several levels. First, IL-6 and S100A9 may complement established clinical risk models such as the IMDC classification by providing biological information about tumor immunobiology and systemic inflammation. Second, they may help guide treatment selection by identifying patients at higher risk who may benefit from combination therapies or closer monitoring. Third, these biomarkers could be used for patient stratification in prospective clinical trials exploring therapies targeting the IL-6 axis, myeloid cells, or tryptophan metabolism. Finally, serial measurement of these biomarkers during treatment may help identify early resistance or treatment failure. However, these applications remain hypothesis-generating and require validation in larger prospective cohorts.

This work has several limitations. The multicenter nature of the study required the shipment of frozen samples, precluding the use of flow cytometry techniques for the analysis of circulating Tregs and MDSCs; therefore, the correlation of these cells with other biomarkers could not be established. Nevertheless, the study shows interesting prognostic patterns and marker correlations; Given the relationships of IL-6 and S100A9 with the antitumor capacities of the immune system, it would be interesting to evaluate the proposed composite marker in patients treated with either TKIs, immunotherapy, or combined treatments, in the context studies with a sufficient number of patients. Taken together, our results suggest that IL-6, S100A9, Arg-1, and tryptophan metabolism are not independent findings but rather components of a common immunometabolic and inflammatory phenotype associated with tumor immune escape, treatment resistance, and poor prognosis in metastatic CCRCC.

## 5 Conclusions

In metastatic CCRCC patients treated with sunitinib, the serum levels of IL-6, Arg-1, and S100A9 have prognostic value, and combination of IL-6 and S100A9 might identify patients at favorable-, intermediate-, and poor-risk. Furthermore, metabolomics analysis revealed significant differences between extreme PFS and OS phenotypes, and significant associations between IL-6 levels and increased tryptophan metabolism, in agreement with increased IDO expression.

## Data Availability

The datasets presented in this study can be found in online repositories (10.5281/zenodo.15322785). The names of the repository/repositories and accession number(s) can be found in the article/supplementary material.

## Glossary

AhR: aryl hydrocarbon receptor
Alb: albumin
Ang-2: angiopoietin-2
Arg-1: arginase-1
CCRCC: clear cell renal cell carcinoma
CT: computed tomography
CTLA-4: cytotoxic T lymphocyte antigen 4
ECOG: Eastern Cooperative Oncology Group
ELISA: Enzyme-Linked Immuno Sorbent Assay
EPT1: Ethanolamine Phosphotransferase 1
ESI: Electrospray ionization
Hb: hemoglobin
HDL: high-density lipoprotein
HMDB: Human Metabolome Data Base
IDO: indoleamine 2,3-dioxygenase
IL-6: interleukin-6
IL-8: interleukin-8
IPA: isopropanol
IMDC: International Metastatic Renal Cell Carcinoma Database Consortium
KAK/STAT: Janus kinase/signal transducer and activator of transcription
KEGG: Kioto Encyclopedia of Genes and Genomes
LCAT: Lecithin-Cholesterol Acyltransferase
LDH: lactate dehydrogenase
LC-MS: liquid chromatography-mass spectrometry
MDSC: myeloid-derived suppressor cell
MMP2: matrix metalloproteinase-2
MS/MS: tandem mass spectrometry
OS: overall survival
PCA: Principal Component Analysis
PD-L1: Programmed Death Ligand 1
PE: phosphatidylethanolamine
PIUMet: Prize-collecting Steiner-forest algorithm for Integrative Analysis of Untargeted Metabolomics
PFS: progression-free survival
QC: Quality Control
QC-SVRC: Quality Control– Support Vector Regression Correction
RCC: renal cell carcinoma
RECIST: Response Evaluation Criteria in Solid Tumors
RSD: Relative standard deviation
RT: retention time
SOCS3: suppressor of cytokine signaling 3
TC: total cholesterol
PDGF: platelet-derived growth factor
PD-1: Programmed cell death protein 1
PFS: progression-free survival
PS: performance status
SOGUG: Spanish Oncology Genitourinary Group
S100A9: S100 calcium binding protein A9
TDO: tryptophan 2,3-dioxygenase
TF: tissue factor
TGFA α: transforming growth factor α
TKI: tyrosine kinase inhibitor
TNF-α: tumor necrosis factor α
VEGF: vascular endothelial growth factor.
